# The influence of delirium on mortality and length of ICU stay and analysis of risk factors for delirium after liver transplantation

**DOI:** 10.3389/fneur.2023.1229990

**Published:** 2023-10-05

**Authors:** Ying Ma, Cuiying Li, Weiting Peng, Qiquan Wan

**Affiliations:** ^1^Department of Transplant Surgery, The Third Xiangya Hospital of Central South University, Changsha, China; ^2^Engineering and Technology Research Center for Transplantation Medicine of National Health Commission, The Third Xiangya Hospital of Central South University, Changsha, China; ^3^Class 2, Grade 2019, 8-Year Clinical Medicine Program, Xiangya School of Medicine, Central South University, Changsha, China

**Keywords:** liver transplantation, delirium, risk factors, prognosis, ICU stay

## Abstract

**Objective:**

To analyze the incidence, timing, risk factors and prognosis of delirium after liver transplantation (LT).

**Methods:**

The clinical data of 321 patients undergoing LT in the Third Xiangya Hospital of Central South University from January 2018 to December 2022 were collected to investigate the incidence, onset, and risk factors for post-LT delirium and the impact of delirium on LT recipients’ prognosis by statistical analysis.

**Results:**

The incidence of post-LT delirium was 19.3% (62/321), and the median interval between LT and onset of delirium was 20.1 h. Univariate analysis showed that pre-LT variables (Model for End Stage Liver Disease (MELD) score, hospital stay, hepatic encephalopathy, infection, white blood cell (WBC) count, lymphocyte count, abnormal potassium, lactulose use), intraoperative variables (red blood cell transfusion, remimazolam use, dexmedetomidine use) and post-LT variables (hypernatraemia, acute rejection, reoperation, basiliximab use, tacrolimus concentration) were associated with post-LT delirium. Multivariate logistic regression analysis revealed that MELD score at LT ≥22 [OR = 3.400, 95% CI:1.468–7.876, *p* = 0.004], pre-LT hepatic encephalopathy [OR = 3.224, 95% CI:1.664–6.244, *p* = 0.001], infection within 2 months prior to LT [OR = 2.238, 95% CI:1.151–4.351, *p* = 0.018], acute rejection [OR = 2.974, 95% CI:1.322–6.690, *p* = 0.008], and reoperation [OR = 11.919, 95% CI:2.938–48.350, *p* = 0.001] were independent risk factors for post-LT delirium. Post-LT delirium was reduced in LT recipients exposing to intraoperative remimazolam [OR = 0.287, 95% CI: 0.113–0.733, *p* = 0.009] or ≥ 25 μg of intraoperative dexmedetomidine [OR = 0.441, 95% CI 0.225–0.867, *p* = 0.018]. As for clinical outcomes, patients with delirium had a higher percentage of staying at the (ICU) ≥7 d after LT than those without delirium [OR = 2.559, 95% CI 1.418–4.617, *p* = 0.002].

**Conclusion:**

The incidence of delirium was high and the onset of delirium was early after LT. Risk factors for post-LT delirium included high MELD score at LT, pre-LT hepatic encephalopathy and infections, acute rejection and reoperation. Intraoperative use of remimazolam or dexmedetomidine reduced post-LT delirium. Delirium had a negative impact on the length of ICU stay.

## Introduction

Delirium is a cognitive disorder that occurs in a short time and involves changes in attention, consciousness, orientation, memory, and perception (1). There are high incidence of postoperative delirium in liver transplant (LT) recipients, ranging from 7.5 to 47% (2–13). Delirium in liver transplant recipients is often associated with metabolic disorders, infection, organ failure, hepatic or uremic encephalopathy, and admission to an intensive care unit (ICU), as well as the neurotoxic side effects from the use of immunosuppressive drugs such as calcineurin inhibitors or high-dose steroids (14–16). Delirium mainly occurs within 1 month after LT, with a median onset time of 2.0–5.5 d postoperatively (2, 4, 6).

Previous studies have shown that various factors can lead to delirium after LT. Preoperative factors include advanced age, high international normalized ratio, hyperbilirubinemia, renal replacement therapy, hepatic encephalopathy, alcohol abuse, alcoholic liver cirrhosis, high Model for End Stage Liver Disease (MELD) score, Child-Turcotte-Pugh classification, acute physiology and chronic health evaluation (APACHE) II score, depression, recent use of antidepressants, and hospitalization. Intraoperative factors include red blood cell (RBC) transfusion, significant blood loss, use of fentanyl, and post-reperfusion syndrome. Postoperative factors include hyperammonemia, high APACHE II score, use of vasopressor drugs or midazolam, mechanical ventilation, reintubation, and prolonged ICU stay and hospitalization (2–6, 8, 9, 11, 13, 17–19).

Some researches have also shown that there are adverse effects on the prognosis for delirium after LT including prolonged postoperative ICU stay, total hospital stay, and mechanical ventilation, increased frequency of blood purification therapy, and mortality, and even long-term cognitive impairment (2, 5, 6, 8, 9, 12, 13). Our present study found that the risk factors for delirium after LT were associated with pre-, intra- and, post-operative variables and delirium was associated with longer ICU stay. Identifying risk factors for delirium after LT could help in early prevention and improve patient outcomes.

## Methods and materials

### General information

A retrospective cohort study was conducted to collect demographic, clinical, and laboratory data from 321 LT recipients of grafts from donation after citizens’ death from January 2018 to December 2022 in The Third Xiangya Hospital of Central South University. All liver grafts, but one which was from circulatory death, were from brain death. There were 264 males and 57 females, with a mean age of 47.1 ± 10.0 years. There were 246 cases with hepatitis virus-related cirrhosis/necrosis/tumor, 20 with alcoholic liver disease, 18 with mixed cirrhosis, 11 with autoimmune hepatitis, 8 with primary biliary cirrhosis, 5 with Budd-Chiari syndrome, 4 with cryptogenic cirrhosis, 3 each with hepatolenticular degeneration and transplanted liver failure, 2 with drug-induced liver injury, and 1 with polycystic liver. Our study was approved by the ethics committee of The Third Xiangya Hospital of Central South University.

### Inclusion and exclusion criteria

We included the adult recipients who underwent LT in our center during the study period, and we excluded the recipients who were under 18 years old, developed infections within 2 weeks before LT, were unconscious and could not be evaluated for delirium, or died during the perioperative period for anesthesia accidents or surgical complications. A total of 321 patients of LT met the inclusion criteria, and 7 patients were excluded from our study, including 1 patient who died of massive bleeding during the operation, 4 patients who were persistent coma after LT, and 2 patients younger than 18 years old.

### Definitions and assessment of delirium

Infection was evaluated according to the criteria of the Centers for Disease Control and Prevention/National Healthcare Safety Network (CDC/NHSN) (20). The cutoff value for the normal serum sodium level in our hospital was defined as 137 and 147 mmol/L. The normal range for serum potassium level in our hospital was 3.5–5.3 mmol/L. Reoperation referred to retransplantation or post-LT laparotomy. Acute rejection is referred to as T cell-mediated rejection or antibody mediated rejection confirmed by pathology. On-duty nurses examined patients for delirium through the ICU Confusion Assessment Method (CAM-ICU) every 8 h after LT and continued for 7 d, or until either discharge from the hospital or death (19, 21). Definitions of post-LT delirium met the diagnostic criteria of delirium in the Diagnostic and Statistical Manual for Mental Disorders, fifth edition (DSM-5) (22). Delirium was defined as an acute episode of neuropsychiatric condition with a fluctuating process of confusion or an altered mental status. Delirium has four characteristics in the CAM-ICU: (1) an acute change in mental status; (2) fluctuations in attention; (3) disordered thinking; and (4) fluctuations in consciousness.

### Treatment strategy

All patients were performed modified piggyback LT under general anesthesia with endotracheal intubation. The initial induction of general anesthesia were propofol (1–1.5 mg/kg), cisatracurium (0.15–0.2 mg/kg), and/or sufentanil (0.07–0.08 μg/kg) and the maintenance were sevoflurane (1%), propofol (2–3 mg/kg/h), remifentanil (5–8 μg/kg/h), and/or cisatracurium (0.05–0.1 mg/kg/h). Dexmedetomidine was given at a dose of 0.5–1 μg/kg when the liver graft was implanted. The bispectral index was maintained at 45–60, and the monitoring count of four successive muscle relaxant stimulations was <3.

Routine cholecystectomy was performed for liver grafts, and a T-tube was placed for biliary drainage in 4 (1.2%) patients. Third-generation cephalosporins or carbapenem antibiotics were used for prophylaxis against Gram-negative bacteria infection during surgery and postoperatively. Prophylaxis against Gram-positive bacteria and fungal infections was given as needed, with a treatment duration of 5–7 d. Immune induction therapy was given to 208 (64.8%) patients with basiliximab, and tacrolimus or ciclosporin A, plus glucocorticoids, was used as the initial immunosuppressive maintenance treatment. The tacrolimus trough concentration was maintained at 8–10 ng/mL within 1 month after LT, then 6–8 ng/mL for months 2–6, and 5–6 ng/mL after 6 months. Methylprednisolone was used as an initial glucocorticoid treatment, and oral prednisone tablets were given on the seventh day. Mycophenolate mofetil or enteric-coated mycophenolate sodium and anti-thymocyte globulin were used if needed. All patients were closely monitored for 3–7 d in the ICU after LT.

### The content and acquisition methods of the data

All of the patients were followed up for 6 months after LT. The data were obtained through electronic medical records, outpatient or telephone follow-up, etc. We included general preoperative, intraoperative, and postoperative conditions of the patients, all demographic, laboratory, and clinical data related to delirium, and postoperative outcomes including survival, ICU length of stay, and length of hospitalization after LT.

### Statistics

We analyzed the data with SPSS 26.0 statistical software package (IBM SPSS Statistics, IBM Corporation, Armonk, NY, USA). Continuous variables were expressed as mean ± standard deviation or median (interquartile range), and categorical data were shown as percentages. Univariate analysis was performed using the Mann–Whitney *U* test, the *t* test, Pearson’s chi-square test or Fisher’s exact test, as appropriate. No missing data were required to handle. There is no collinearity between these 16 variables associated with post-LT delirium with all variance inflation factor < 2. Variables with statistically significant differences in univariate analysis were introduced into the final multivariate model, and performed using binary logistic regression based on forward stepwise logistic regression. The association was expressed as an odds ratio (OR) value and 95% confidence interval (CI) to describe the independent factors related to delirium. A two-tailed *p* value of <0.05 was considered statistically significant.

## Results

### General characteristics and prognosis of LT recipients

A total of 321 patients were eventually included in this retrospective study. There were 264 (82.2%) males with a mean age of 47.1 ± 10.0 years. A total of 62 (19.3%) patients developed delirium with a median onset time of 20.1 h after LT. Of these patients, 36 (58.1%) developed delirium within 24 h after LT. The median MELD score at LT was 24. A total of 157 (48.9%) patients had infections within 2 months before LT including 128 (39.9%) patients with pulmonary infections and 19 (5.9%) patients with multiple sites infections, and all these 19 patients had pulmonary infections. The primary liver diseases were mainly hepatitis viral-related cirrhosis/necrosis/tumor (246, 76.7%), and alcoholic cirrhosis (20, 6.2%). There were 91 (28.3%) patients with hepatic encephalopathy before LT. It was 0.8 mg/dL, 3.9 mmol/L, 138.2 mmol/L, 34.2 g/L, 5.3 × 10^9^/L, 0.8 × 10^9^/L, and 67.0 × 10^9^/L for the median pre-LT creatinine, potassium, sodium, albumin, WBC count, lymphocyte count, and platelet count, respectively.

Eighty-one (25.2%) and 238 (74.1) patients received remimazolam with the maximal dosage 114 mg and dexmedetomidine with the maximal dosage 200 μg during surgery, respectively. It was 0 (0–5.0) mg, 2 (0–2.0) mg, 30.0 (0–50.0) μg and 850.0 (550.0–1085.0) mg for the median (interquartile range) dosage of remimazolam, midazolam, dexmedetomidine and propofol use, respectively. And it was 375.0 min for the median duration of LT, while the median blood loss was 3000.0 mL and a median RBC transfusion was 12.0 units.

Before opening the portal vein of the liver graft, all patients received 500 mg methylprednisolone and 208 (64.8%) patients received basiliximab for immune induction therapy. After LT, 17 patients (5.3%) were treated with anti-thymocyte immunoglobulin therapy, 318 (99.1%) with tacrolimus. The median ALT, creatinine, albumin, potassium, and sodium on day 1 after LT were 697.0 U/L, 1.1 mg/dL, 37.2 g/L, 3.7 mmol/L, and 145.0 mmol/L, respectively. After LT, 41 patients required mechanical ventilation, 45 developed acute rejection and 13 underwent reoperation. The median length of ICU stay was 6.0 d after LT. A total of 17 patients died within 1 month, 20 within 2 months, and 23 within 6 months after LT (Table 1).

**Table 1:** Demographic, laboratory, and clinical variables of 321 LT recipients.

Characteristics	Value
Recipient age, mean years ± SD	47.1 ± 10.0
Recipient gender, no. of male (%)	264 (82.2)
Recipient BMI, median (IQR), kg/m^2^	22.8 (20.8–25.4)
Hospital stay prior to LT, median (IQR), days	10 (1.0–25.0)
MELD score at LT, median (IQR)	24 (15.0–30.0)
Infection within 2 months prior to LT, *n* (%)	157 (48.9)
Pulmonary infection	128 (39.9)
Abdominal/biliary infection	8 (2.5)
Bloodstream infection	1 (0.3)
Urinary tract infection	1 (0.3)
Multiple site infection^1^	19 (5.9)
Underlying liver diseases, *n* (%)	
Viral cirrhosis/necrosis/tumor	246 (76.7)
Alcoholic cirrhosis	20 (6.2)
Autoimmune hepatitis	11 (3.4)
Primary biliary cirrhosis	8 (2.5)
Mixed cirrhosis	18 (5.6)
Others^2^	18 (5.6)
Pre-LT hepatic encephalopathy, *n* (%)	91 (28.3)
Pre-LT type 2 diabetes, *n* (%)	38 (11.8)
Pre-LT use of lactulose, *n* (%)	53 (16.5)
Pre-LT creatinine, median (IQR), mg/dL	0.8 (0.6–1.0)
Pre-LT WBC count, median (IQR), ×10^9^/L	5.3 (3.5–8.2)
Pre-LT lymphocyte count, median (IQR), ×10^9^/L	0.8 (0.5–1.2)
Pre-LT platelet count, median (IQR), ×10^9^/L	67 (42.0–101.5)
Pre-LT albumin level, median (IQR), g/L	34.2 (30.7–37.5)
Pre-LT potassium, median (IQR), mmol/L	3.9 (3.6–4.3)
Pre-LT sodium, median (IQR), mmol/L	138.2 (135.4–141.3)
Donor age, mean years ± SD	44.3 ± 12.5
Donor BMI, median (IQR), kg/m^2^	23 (21.3–25.2)
Steatosis ≥ 10%, *n* (%)	137 (42.7)
Cold ischemia time, mean h ± SD	6.3 ± 1.6
Duration of surgery, median (IQR), min	375.0 (335.0–424.5)
Intraoperative bleeding, median (IQR), mL	3000.0 (2000.0–4500.0)
Intraoperative RBC transfusion, median (IQR), units	12.0 (8.0–17.0)
Intraoperative use of midazolam, median (IQR), mg	2.0 (0–2.0)
Intraoperative use of remimazolam, *n* (%)	81 (25.2)
Intraoperative use of propofol, median (IQR), mg	850.0 (550.0–1085.0)
Intraoperative use of dexmedetomidine, median (IQR), μg	30.0 (0–50.0)
Preoperative and intraoperative imipenem/Cilastatin use, *n* (%)	90 (28.0)
Post-LT immunosuppressant treatment, *n* (%)	
Tacrolimus	318 (99.1)	Characteristics	Value
Ciclosporin A	2 (0.6)
Mycophenolate mofetil/enteric-coated mycophenolate sodium	247 (76.9)
Sirolimus	3 (0.9)
Glucocorticoid	321 (100)
Basiliximab	208 (64.8)
Anti-thymocyte globulin	17 (5.3)
ALT on day 1 after LT, median (IQR), U/L	697.0 (396.0–1330.5)
Creatinine on day 1 after LT, median (IQR), mg/dL	1.1 (0.8–1.4)
Albumin level on day 1 after LT, median (IQR), g/L	37.2 (33.8–40.4)
Potassium on day 1 after LT, median (IQR), mmol/L	3.7 (3.4–4.1)
Sodium on day 1 after LT, median (IQR), mmol/L	145.0 (142.0–147.0)
Tacrolimus on day 5 after LT, median (IQR), ng/dL	6.0 (4.6–8.0)
Post-LT mechanical ventilation, *n* (%)	41 (12.8)
Reoperation, *n* (%)	13 (4.0)
Post-LT infection, *n* (%)	163 (50.8)
Acute rejection, *n* (%)	45 (14.0)
Post-LT renal replacement therapy, *n* (%)	10 (3.1)
ICU stay after LT, median (IQR), days	6.0 (5.0–7.0)
Hospitalization stay after LT, median (IQR), days	26.0 (22.0–30.0)
Delirium cases after LT, *n* (%)	62 (19.3)
Delirium onset time from LT, median (IQR), h	20.1 (5.2–72.6)
All-cause mortality within 1 month after LT, *n* (%)	17 (5.3)
All-cause mortality within 2 months after LT, *n* (%)	20 (6.2)
All-cause mortality within 6 months after LT, *n* (%)	23 (7.2)

ALT, alanine aminotransferase; BMI, body mass index; ICU, intensive care unit; IQR, interquartile range; LT, liver transplantation; MELD, Model for End-Stage Liver Disease; RBC, red blood cell; SD, standard deviation; WBC, white blood cell.

^1^There were 8 cases of pulmonary and abdominal infections, 6 cases of pulmonary, abdominal, and bile duct infections, 3 cases of pulmonary and bile duct infections, and 1 case each of pulmonary, abdominal, and bloodstream infections, as well as pulmonary and intracranial infections.

^2^There were 5 cases of Budd-Chiari syndrome, 4 cases of cryptogenic cirrhosis, 3 cases each of hepatolenticular degeneration and transplant liver failure, 2 cases of drug-induced liver injury, and 1 case of polycystic liver.

### Analysis of the risk factors for delirium after LT

The results of the univariate analysis showed that some factors were associated with post-LT delirium, including MELD score at LT ≥22 (*p* < 0.001), pre-LT hospitalization ≥7 d (*p* = 0.008), pre-LT use of lactulose (*p* = 0.001), pre-LT hepatic encephalopathy (*p* < 0.001), pre-LT infection cases within 2 months (*p* = 0.002), pre-LT WBC count ≥10 × 10^9^/L (*p* < 0.001), pre-LT lymphocyte count ≤0.5 × 10^9^/L (*p* = 0.018), pre-LT abnormal potassium (*p* = 0.035), intraoperative RBC transfusion ≥8 U (*p* = 0.024), intraoperative remimazolam use (*p* = 0.012), intraoperative dexmedetomidine use ≥25 μg (*p* = 0.015), hypernatraemia on day 1 after LT (*p* = 0.001), tacrolimus on day 5 after LT ≥ 10 ng/dL (*p* = 0.017), reoperation (*p* = 0.012), acute rejection (*p* = 0.003), and basiliximab use≥40 mg (*p* = 0.019) (Table 2).

**Table 2:** Univariate analysis of risk factors for delirium in LT recipients.

Variables	Delirium (62)	No Delirium (259)	*χ^2^*	*p* value
Male sex, *n* (%)	50 (80.6)	214 (82.6)	0.116	0.734
Age ≥ 55 years, *n* (%)	17 (27.4)	56 (21.6)	0.957	0.328
Recipient BMI ≥ 25	14 (22.6)	75 (28.8)	0.933	0.334
MELD score at LT ≥ 22, *n* (%)	54 (87.1)	137 (52.9)	23.737	<0.001
Hospital stay prior to LT ≥ 7 days, *n* (%)	46 (74.2)	144 (55.6)	7.120	0.008
Viral cirrhosis/necrosis/tumor, *n* (%)	49 (79.0)	197 (76.1)	0.247	0.620
Alcoholic cirrhosis, *n* (%)	3 (4.8)	17 (6.6)	0.045	0.832
Pre-LT diabetes, *n* (%)	7 (11.3)	31 (12.0)	0.028	0.866
Pre-LT use of lactulose, *n* (%)	19 (30.6)	34 (13.1)	11.136	0.001
Pre-LT creatinine ≥ 2 mg/dL, *n* (%)	3 (4.8)	15 (5.8)	0	1.000
Pre-LT hepatic encephalopathy, *n* (%)	32 (51.6)	59 (22.8)	20.117	<0.001
Infection within 2 months prior to LT, *n* (%)	41 (66.1)	116 (44.8)	9.478	0.002
Pre-LT WBC count ≥ 10 × 10^9^/L, *n* (%)	19 (30.6)	23 (8.9)	17.875	<0.001
Pre-LT lymphocyte count ≤ 0.5 × 10^9^/L, *n* (%)	7 (11.3)	66 (25.5)	5.603	0.018
Pre-LT platelet count ≤ 50 × 10^9^/L, *n* (%)	26 (41.9)	91 (35.1)	1.032	0.310
Pre-LT albumin level < 30 g/L, *n* (%)	12 (19.4)	53 (20.5)	0.049	0.824
Pre-LT abnormal potassium, *n* (%)	20 (32.3)	51 (19.7)	4.448	0.035
Pre-LT hypernatraemia, *n* (%)	4 (6.5)	9 (3.5)	0.503	0.478
Donor age ≥ 50 years	21 (33.9)	103 (39.6)	0.771	0.380
Donor BMI ≥ 25 kg/m^2^, *n* (%)	14 (22.6)	78 (30.0)	1.389	0.239
Steatosis ≥ 30%, *n* (%)	4 (6.5)	30 (11.5)	1.391	0.238
Cold ischemia time ≥ 360 min, *n* (%)	30 (48.4)	131 (50.6)	0.096	0.756
Duration of surgery ≥ 400 min, *n* (%)	25 (40.3)	92 (35.5)	0.521	0.470
Intraoperative bleeding ≥ 3,000 mL, *n* (%)	34 (54.8)	143 (55.2)	0.003	0.953
Intraoperative RBC transfusion ≥ 8 U, *n* (%)	55 (88.7)	195 (75.3)	5.099	0.024
Intraoperative use of midazolam, *n* (%)	44 (71.0)	152 (58.5)	1.052	0.305
Intraoperative remimazolam use, *n* (%)	8 (12.9)	73 (28.2)	6.336	0.012
Intraoperative dexmedetomidine use ≥ 25 μg, *n* (%)	35 (56.5)	188 (72.6)	5.950	0.015
Intraoperative propofol use≥1,000 mg, *n* (%)	17 (27.4)	90 (34.7)	1.171	0.279
Preoperative and intraoperative Imipenem/Cilastatin use, *n* (%)	18 (20.9)	72 (27.8)	0.025	0.873
ALT on day 1 after LT ≥ 1,000 U/L, *n* (%)	24 (38.7)	83 (32.0)	1.042	0.307
Creatinine on day 1 after LT ≥ 2 mg/dL, *n* (%)	11 (17.7)	25 (9.7)	3.204	0.073
Albumin level on day 1 after LT < 30 g/L, *n* (%)	4 (6.5)	17 (6.6)	0	1.000
Abnormal potassium on day 1 after LT, *n* (%)	23 (37.1)	68 (26.3)	2.772	0.096
Hypernatraemia on day 1 after LT, *n* (%)	24 (38.7)	49 (18.9)	10.922	0.001
Tacrolimus on day 5 after LT ≥ 10 ng/dL	12 (19.4)	23 (8.8)	5.650	0.017
Post-LT mechanical ventilation, *n* (%)	11 (17.7)	30 (11.5)	0.743	0.389
Reoperation, *n* (%)	6 (9.7)	7 (2.7)	6.263	0.012
Post-LT infection, *n* (%)	37 (59.7)	126 (48.5)	1.396	0.237
Acute rejection, *n* (%)	16 (25.8)	29 (11.2)	8.858	0.003
Post-LT renal replacement therapy, *n* (%)	4 (6.5)	6 (2.3)	1.449	0.229
Glucocorticoidse ≥ 1,500 mg, *n* (%)	36 (58.1)	145 (55.8)	1.42	0.233
Basiliximab use ≥ 40 mg, *n* (%)	22 (35.5)	135 (51.9)	5.543	0.019

ALT, alanine aminotransferase; BMI, body mass index; LT, liver transplantation; MELD, Model for End-Stage Liver Disease; RBC, red blood cell; WBC, white blood cell.

We further performed multivariate analysis and the results showed that MELD score at LT ≥22 [OR = 3.400, 95% CI:1.468–7.876, *p* = 0.004], pre-LT hepatic encephalopathy [OR = 3.224, 95% CI:1.664–6.244, *p* = 0.001], infection within 2 months prior to LT [OR = 2.238, 95% CI:1.151–4.351, *p* = 0.018], acute rejection [OR = 2.974, 95% CI:1.322–6.690, *p* = 0.008], and reoperation [OR = 11.919, 95% CI:2.938–48.350, *p* = 0.001] were independent risk factors for post-LT delirium. Post-LT delirium was reduced in LT recipients exposing to intraoperative remimazolam [OR = 0.287, 95% CI: 0.113–0.733, *p* = 0.009] or ≥ 25 μg of intraoperative dexmedetomidine [OR = 0.441, 95% CI 0.225–0.867, *p* = 0.018] (Table 3). The goodness of fit test showed a good result with a *p* value greater than 0.05 using Hosmer-Lemeshow test.

**Table 3:** Multivariate logistic regression analysis of risk factors for delirium in LT recipients.

Variables	*B*	S.E.	Wald*χ^2^*	OR (95% CI)	*p* value
MELD score at LT ≥ 22	1.224	0.429	8.152	3.400 (1.468–7.876)	0.004
Pre-LT hepatic encephalopathy	1.170	0.337	12.043	3.224 (1.664–6.244)	0.001
Infection within 2 months prior to LT	0.806	0.339	5.639	2.238 (1.151–4.351)	0.018
Acute rejection	1.090	0.414	6.939	2.974 (1.322–6.69)	0.008
Reoperation	2.478	0.714	12.030	11.919 (2.938–48.350)	0.001
Intraoperative dexmedetomidine use≥25 μg	−0.818	0.344	5.636	0.441 (0.225–0.867)	0.018
Intraoperative remimazolam use	−1.247	0.478	6.810	0.287 (0.113–0.733)	0.009

LT, liver transplant; MELD, Model for End-Stage Liver Disease; WBC, white blood cell.

### Prognosis of patients with delirium after LT

Our result revealed that more patients with delirium stayed at ICU ≥7 d after LT than those without delirium (*p* < 0.001). Delirium seems to increase mortality within 1 month (*p* = 0.019) after LT, but have no effects on mortality within 2 months (*p* = 0.067) and 6 months (*p* = 0.161) after LT compared to non-delirium (Table 4).

**Table 4:** Postoperative outcome for patients with/without delirium following LT.

Variables	Delirium (62)	No delirium (259)	*χ^2^*	*p* value
ICU stay after LT ≥ 7 days, *n* (%)	30 (48.4)	69 (26.6)	13.224	<0.001
Hospitalization stay after LT ≥ 21 days, *n* (%)	56 (90.3)	211 (81.5)	2.804	0.094
All-cause mortality within 1 month after LT, *n* (%)	7 (11.3)	10 (3.9)	5.505	0.019
All-cause mortality within 2 month after LT, *n* (%)	7 (11.3)	13 (5.0)	3.367	0.067
All-cause mortality within 6 month after LT, *n* (%)	7 (11.3)	16 (6.2)	1.966	0.161

ICU, intensive care unit; LT, liver transplantation.

In order to decide if delirium was an independent risk factor for early mortality, we performed an analysis of those factors including delirium that might be associated with mortality and verified that recipient age ≥ 55 years [OR = 3.422, 95% CI:1.187–9.980, *p* = 0.023], post-LT infection [OR = 13.546, 95% CI:1.744–105.206, *p* = 0.013] and post-LT renal replacement therapy [OR = 9.477, 95% CI:2.035–44.131, *p* = 0.004], not delirium, were independently associated with mortality within 1 month after LT (Table 5). There is no collinearity between these 6 variables associated with all-cause mortality within 1 month after LT with all variance inflation factor < 2.

**Table 5:** Univariate and multivariate logistic regression analysis of risk factors for all-cause mortality within 1 month after LT.

Variables	Death (17)	Survival (304)	*p*	OR (95% CI)
Total, *n* (%)				
Univariate analysis				
Male sex, *n* (%)	10 (58.8)	254 (83.6)	0.010	
Recipient age ≥ 55 years, *n* (%)	9 (52.9)	64 (21.1)	0.002	
Alcoholic cirrhosis, *n* (%)	0 (0)	29 (9.5)	0.368	
Pre-LT diabetes, *n* (%)	2 (11.8)	38 (11.8)	1.000	
Infection within 2 months prior to LT, *n* (%)	11 (64.7)	146 (48.0)	0.185	
Pre-LT hepatic encephalopathy, *n* (%)	7 (41.2)	84 (27.6)	0.231	
MELD score at LT ≥22, *n* (%)	13 (76.5)	178 (58.6)	0.143	
Steatosis≥30%, *n* (%)	2 (11.8)	32 (10.5)	1.000	
Duration of surgery≥400 min, *n* (%)	11 (64.7)	106 (34.9)	0.013	
Intraoperative bleeding≥3,000 mL, *n* (%)	13 (76.5)	164 (53.9)	0.071	
Intraoperative RBC transfusion≥8 U, *n* (%)	14 (82.4)	236 (77.6)	0.876	
ALT on day 1 after LT ≥ 1,000 U/L, *n* (%)	9 (52.9)	107 (33.2)	0.074	
Albumin level on day 1 after LT < 30 g/L, *n* (%)	3 (17.6)	18 (5.9)	0.162	
Delirium, *n* (%)	7 (41.2)	55 (18.1)	0.019	
Acute rejection, *n* (%)	1 (5.9)	44 (14.5)	0.526	
Reoperation, *n* (%)	2 (11.8)	11 (3.6)	0.168	
Post-LT infection, *n* (%)	16 (94.1)	147 (48.2)	<0.001	
Post-LT renal replacement therapy, *n* (%)	4 (23.5)	6 (2.0)	0.001	
Multivariate analysis				
Recipient age ≥ 55 years			0.023	3.422 (1.187–9.980)
Post-LT infection			0.013	13.546 (1.744–105.206)
Post-LT renal replacement therapy			0.004	9.477 (2.035–44.131)

ALT, alanine aminotransferase; CI, confidence intervals; LT, liver transplant; MELD, Model for End-Stage Liver Disease; OR, odds ratios; RBC, red blood cell.

However, when we performed an analysis of the factors related to prolonged ICU stay and found that delirium was independently associated with ICU stay after LT ≥ 7 days, among other variables such as creatinine on day 1 after LT ≥ 1.5, post-LT infection, and post-LT mechanical ventilation (Table 6).

**Table 6:** Univariate and multivariate logistic regression analysis of risk factors for ICU stay after LT ≥ 7 days.

Variables	ICU stay ≥7 d (99)	ICU stay <7 d (222)	*p*	OR (95% CI)
Total, *n* (%)				
Univariate analysis				
Male sex, *n* (%)	76 (76.8)	188 (84.7)	0.086	
Age ≥ 55 years, *n* (%)	24 (24.2)	49 (22.1)	0.668	
Pre-LT diabetes, *n* (%)	11 (11.1)	27 (12.2)	0.788	
Hospital stay prior to LT ≥7 days, *n* (%)	68 (68.7)	122 (55.0)	0.021	
Infection within 2 months prior to LT, *n* (%)	50 (50.5)	107 (48.2)	0.703	
Pre-LT hepatic encephalopathy, *n* (%)	36 (36.4)	55 (24.8)	0.033	
MELD score at LT ≥ 22, *n* (%)	73 (73.7)	118 (53.2)	0.001	
Pre-LT WBC count≥10 × 10^9^/L, *n* (%)	17 (17.2)	25 (11.3)	0.147	
Pre-LT lymphocyte count ≤ 0.5 × 10^9^/L, *n* (%)	20 (20.2)	53 (23.9)	0.469	
Pre-LT platelet count ≤ 50 × 10^9^/L, *n* (%)	34 (34.3)	83 (37.4)	0.601	
Steatosis ≥ 30%, *n* (%)	10 (10.1)	24 (10.8)	0.839	
Duration of surgery ≥ 400 min, *n* (%)	41 (41.4)	76 (34.2)	0.217	
Intraoperative bleeding ≥ 3,000 mL, *n* (%)	63 (63.6)	114 (51.4)	0.045	
Intraoperative RBC transfusion ≥ 8 U, *n* (%)	78 (78.8)	172 (77.5)	0.794	
ALT on day 1 after LT ≥ 1,000 U/L, *n* (%)	38 (38.4)	69 (31.1)	0.181	
Albumin level on day 1 after LT < 30 g/L, *n* (%)	10 (10.1)	11 (5.0)	0.085	
Creatinine on day 1 after LT ≥ 1.5 mg/dL, *n* (%)	31 (31.3)	39 (17.6)	0.006	
Post-LT mechanical ventilation, *n* (%)	21 (21.2)	20 (9.0)	0.002	
Delirium, *n* (%)	31 (31.3)	31 (14.0)	<0.001	
Acute rejection, *n* (%)	13 (13.1)	32 (14.4)	0.760	
Reoperation, *n* (%)	7 (7.1)	6 (2.7)	0.067	
Post-LT infection, *n* (%)	63 (63.6)	100 (45.0)	0.002	
Multivariate analysis				
Delirium			0.002	2.559 (1.418–4.617)
Creatinine on day 1 after LT ≥ 1.5 mg/dL			0.024	1.941 (1.091–3.452)
Post-LT infection			0.016	1.868 (1.124–3.105)
Post-LT mechanical ventilation			0.029	2.191 (1.085–4.422)

ALT, alanine aminotransferase; CI, confidence intervals; ICU, intensive care unit; LT, liver transplant; MELD, Model for End-Stage Liver Disease; OR, odds ratios; RBC, red blood cell; WBC, white blood cells.

## Discussion

The incidence of delirium after LT is high, but there are great differences among different studies, which is related to the variations in the diagnosis methods of delirium and the proportions of patients with alcoholic liver disease and hepatic encephalopathy (4). In our study, the incidence of delirium was 19.3 and 58.1% of them occurred within 24 h after LT, which was close to the incidence (17%) and the time of onset (88% on the day or the first day after surgery) reported by Lee et al. (4) and was extremely closely to the post-LT incidence (19.4%) reported by Lu et al. (19).

MELD score, which represents the severity of liver disease, affects the priority allocation of the liver and predicts the mortality of patients preparing for LT (23). The results of our study showed that a high MELD score at LT led to an increased incidence of delirium after LT, which was consistent with other studies (4, 8, 11, 13).

Our study also found that pre-LT infection within 2 months, not post-LT infection, was associated with the onset of delirium. Wang SH et al. and Bhattacharya et al. showed that infection after LT increased the occurrence of post-LT delirium (2, 13). However, whether infection is a cause or a consequence of delirium remains elusive, though infection is well-proven risk factors for delirium, especially pneumonia and urinary tract infection (13).

In our study, the results showed that pre-LT hepatic encephalopathy was associated with the occurrence of post-LT operative delirium, which was consistent with the results of several studies (3, 9, 11, 12). Patients with liver disease who underwent surgery and were admitted to the ICU were prone to delirium. The reason might be that liver diseases can affect the brain, in which activation of microglia cells plays a key role in leading to nervous system inflammation. In addition, the occurrence of delirium is also related to changes in astrocyte morphology, brain metabolism, brain perfusion, and blood–brain barrier permeability which were caused by hepatic encephalopathy (14, 24–26).

Some studies showed that it was prone to causing delirium for benzodiazepines such as midazolam, lorazepam, and propofol, while there was no correlation between midazolam, propofol and delirium in our study (8). Our study did not find the relationship between midazolam and delirium. The reason was that the amount (median dosage 2 mg) of midazolam used in all LT recipients was minimal, which could be insufficient to cause delirium. We found that dexmedetomidine reduced the occurrence of delirium. Dexmedetomidine, compared with benzodiazepines, was associated with less onset and shorter duration of delirium (27). Some research showed that dexmedetomidine might play a neuroprotective role by reducing the release of inflammatory mediators and neuroendocrine hormones, and better maintaining intracranial homeostasis (28).

Interestingly, we also found intraoperative use of remimazolam, one kind of benzodiazepines, reduced the occurrence of delirium. The finding was in line with a study reporting that in children undergoing tonsillectomy and adenoidectomy, the use of remimazolam led to a significantly lower delirium, compared with 0.9% saline (29). Another stduy revealed that remimazolam reduced the incidence of delirium after transcatheter aortic valve implantation under general anesthesia (30). Deng et al. claimed that both remimazolam and dexmedetomidine were equally effective at acutely mitigating postoperative delirium in older patients after orthopedic surgery with remimazolam having a longer time to delirium resolution than dexmedetomidine (31). The effect of remimazolam on delirium remains to be well elucidated but is of obvious interest. Specifically, we look forward to future studies addressing its effect on post-LT delirium.

Our findings also revealed that acute rejection and reoperation were two risk factors for delirium. Acute rejection means more immunosuppressant use and more infections occur, which can make delirium more likely to occur. Reoperation means more blood transfusions and more sedatives, which can also lead to delirium. At present, there are no reports of increased delirium caused by acute rejection and reoperation after LT. Whether they cause delirium needs to be confirmed by more studies, and the mechanism needs to be further explored in the future.

Lescot et al. and Zhou et al. reported that the amount of RBC infusion correlated with delirium after LT (6, 18). In our study, univariate analysis found that patients with intraoperative RBC infusion ≥8 U were more prone to develop postoperative delirium, but this statistical difference was not maintained in subsequent multivariate analysis. These results indicated that RBC infusion had less effect on delirium than other factors such as hepatic encephalopathy and high MELD score.

Electrolyte imbalance (sodium or potassium) was one of the most common risk factors for postoperative delirium (7). Hackworth et al. also claimed that pre-LT abnormal serum sodium was associated with an increased risk of post-LT delirium (32). Our present study found hypernatraemia on day 1 after LT was associated with post-LT delirium in univariate analysis but did remain as a significant risk factor for delirium in multivariate analysis.

In a previous publication, donor factors, such as graft macrovesicular steatosis, emerged as possibly associated with the likelihood of post-LT delirium (33). We inluded recipient age and body mass index, and steatosis of liver graft into the analysis and did not find their relationship with delirium.

The relationship between delirium and mortality is controversial. Several studies showed that delirium did not affect in-hospital or 1 year mortality with proper treatment (4, 34, 35). Nevertheless, Lescot et al. and Wang et al. reported that delirium after LT was related to in-hospital and 1 year mortality (2, 6). Beckmann et al. also found that patients with post-LT delirium had a shorter mean survival time than those without (12). In Oliver’s study, LT recipients with delirium were more likely to have higher 6 month mortality than patients who did not develop delirium (9). In univariate analysis, we found that patients with delirium had an increased mortality rate within 1 month after LT but had no effects on mortality within 2 and 6 months after LT compared with patients without delirium. However, when performed an analysis of these factors including delirium that might be associated with mortality, we verified that advanced recipient age, post-LT infection and renal replacement therapy, not delirium, were independently associated with mortality within 1 month after LT. There is no collinearity between those 6 variables associated with all-cause mortality within 1 month after LT with all variance inflation factor < 2. So delirium was not an independent risk factor for all-cause mortality within 1 month after LT.

Our study also investigated the effect of delirium on ICU and hospital stay after LT and found that delirium, among other variables such as creatinine on day 1 after LT ≥ 1.5, post-LT infection, and post-LT mechanical ventilation, was associated a longer time of postoperative ICU stay, consistent with previous reports (4, 6, 9, 13, 34, 36). We did not demonstrate a significant difference in postoperative length of hospital stay between patients with and without delirium, in line with the study from Chen et al. (36).

The pathophysiological mechanisms of delirium are far from fully understood (37). Considering the negative effect of delirium on outcomes and no single intervention or medication for treatment, early identification of correctable risk factors for delirium after LT could help treat it with multiple strategies.

Enhanced Recovery After Surgery (ERAS) is a multimodal and perioperative management pathway to minimize the severity of the surgical stress and to decrease postoperative complications (38). The use of ERAS program, which in part aim to minimize opioid exposure, have been related to reduced postoperative delirium among cardiac surgery ICU patients (39). ERAS has not yet been widely accepted in LT recipients, although studies verified that ERAS is safe and effective in accelerating discharge, shortening the duration of LT operation and the anhepatic period of the surgery, reducing intraoperative blood loss and blood transfusion rate, and shortening the ICU and hospital stays without increasing the incidence of complications, readmission and mortality, even reducing the incidence of total complications or severity of complications (40–45). Recommendations from the 2022 International Liver Transplantation Society consensus conference addressed the effect of short term outcomes on survival of both LT recipients and liver grafts, a clear trend toward lower survival in the presence of short-term complications which can be reduced potentially through elements of ERAS (46). Until now, there has been no studies on the effect of ERAS on the occurrence of post-LT delirium. Therefore, prospective trials are required to confirm the item in future.

In our study, we identified 5 risk factors and 2 protective factors for delirium after LT. These factors were classified into two categories. One was related to the severity and complexity of the disease including high MELD score at LT, pre-LT hepatic encephalopathy, infection within 2 months prior to LT, acute rejection and reoperation, and the other was related to the use of sedative drugs, including the intraoperative use of remimazolam and dexmedetomidine. Further research is still required to confirm the extent of the impact of these factors on delirium and to clarify their specific mechanisms.

## Data availability statement

The original contributions presented in the study are included in the article/supplementary material, further inquiries can be directed to the corresponding author.

## Ethics statement

The studies involving humans were approved by the Ethics Committee of The Third Xiangya Hospital of Central South University (NO. 23607). The studies were conducted in accordance with the local legislation and institutional requirements. The ethics committee/institutional review board waived the requirement of written informed consent for participation from the participants or the participants’ legal guardians/next of kin because it is a retrospective cohort study.

## Author contributions

QW conceived and designed the study. YM and CL analyzed and interpreted the data. QW, YM, and CL drafted the manuscript and had full access to all of the data in the study. All authors acquired the data, reviewed the manuscript and vouch for the accuracy and completeness of the data and for the adherence of the study to the protocol, and contributed to the article and approved the submitted version.
